# Patterns of use, dosing, and economic impact of biologic agent use in patients with rheumatoid arthritis: a retrospective cohort study

**DOI:** 10.1186/1471-2474-5-36

**Published:** 2004-10-14

**Authors:** Thomas D Gilbert, Daniel Smith, Daniel A Ollendorf

**Affiliations:** 1 Market Research, The Ipsen Group, Inc., 27 Maple Street, Milford, MA 01757, USA; 2 Analytic Operations, PharMetrics, Inc., 311 Arsenal Street, Watertown, MA 02472, USA

## Abstract

**Background:**

Variability in dosing and costs of biologics among patients with rheumatoid arthritis (RA) is of interest to healthcare descision-makers. We examined dosing and costs among RA patients newly treated with infliximab or etanercept under conditions of typical clinical practice.

**Methods:**

Integrated pharmacy and medical claims data were obtained from 61 U.S. health plans. RA patients newly treated with infliximab or etanercept between July 1999–June 2002 were selected. A maintenance number of infliximab vials was determined after the "loading period" (2–3 infusions); those with ≥ 2 occurrences of an increase in vials or an interval between infusions of <49 days were considered to have had escalated. For etanercept patients, escalation was based on ≥ 2 instances of increased average daily dose. Multiple logistic regression analyses were conducted to assess variables associated with dose escalation. RA-related costs at one year post-initiation also were examined; comparisons were made using generalized linear models.

**Results:**

A total of 1,548 patients were identified (n = 598 and 950 for infliximab and etanercept respectively). Infliximab recipients were somewhat older (50.5 vs. 46.6 years for etanercept). Nearly 60% of infliximab patients increased their dose at one year, compared to 18% for etanercept. Infliximab patients who escalated dose incurred a 25% increase in mean one-year costs ($20,915 vs. $16,713 for no increase; p < 0.0001). Costs among etanercept patients did not substantially differ based on dose escalation ($14,482 vs. $13,866 respectively).

**Conclusions:**

Infliximab is associated with higher rates of dose escalation relative to etanercept, which contributes to substantially higher one-year medical costs.

## Background

Rheumatoid arthritis is a costly and debilitating autoimmune disorder that is characterized by joint pain, stiffness, and impaired functionality. Symptoms arise from the inflammation and degradation of the synovial membrane, causing progressive disability in joint function [1]. As the disease progresses, patients require more frequent invasive procedures (e.g., joint injections, synovectomy) as well as the eventual replacement of affected joints. Consequently, the economic costs of RA are considerable, as the estimated direct and indirect costs of related care in the US totals $19 billion annually [2].

Because there is no known cure for RA, the goal of therapy is to treat the disease's symptomatology while attempting to slow or halt its overall progression. Pharmacotherapy is the cornerstone of treatment where symptoms may be treated with various combinations of nonsteroidal anti-inflammatory drugs (NSAIDs), corticosteroids, and narcotic analgesics. In addition, disease-modifying antirheumatic drugs (DMARDs) are sometimes administered in an effort to alter the disease's progression. The effectiveness of DMARDs, however, is offset by the high levels of toxicity experienced by some patients taking these medications, and is problematic for long-term therapy [3].

In addition to the use of various DMARDs either alone or in combination with other therapies, several new DMARDs have recently been introduced for the treatment of RA. These include two biologic agents, etanercept (Enbrel^®^, Amgen, Inc./Wyeth, Inc., Thousand Oaks, CA and St. David's, PA) and infliximab (Remicade^®^, Centocor Inc., Malvern, PA) which provide anti-rheumatic activity by inhibiting tumor necrosis factor (TNF), another important mediator of an inflammatory response. The use of such agents in combination with the DMARD methotrexate has been shown to be clinically superior to methotrexate alone in controlled clinical trials [4-12].

The results of recent observational studies examining the effectiveness of infliximab indicate that increased or more frequent dosing (i.e., beyond what is mentioned in the product labeling) may provide additional benefits for patients with rheumatoid arthritis [13,14]. However, the costs associated with use of biologic agents is already far greater than other DMARDs. Therefore, economic considerations may impact physicians' willingness to prescribe as well as commercial insurers' placement of biologic agents in the sequence of care.

Given the wide disparity in costs between therapy alternatives and the potential impact of dose escalation on these costs, it is essential to examine the total costs of RA-related care associated with each form of therapy from a perspective of typical U.S. clinical practice. In this study, the direct costs of RA-related care were estimated on an annual basis after initial treatment with anti-TNF biologics. Resource utilization and cost estimates were also stratified by dosing status (increase in dose during follow-up vs. no increase), using retrospective claims data from commercial insurers in the US.

## Methods

### Data source

Medical and pharmaceutical service claims were obtained from the PharMetrics Patient-Centric Database, and spanned the period from January 1999 to June 2002. At the time of this study, the database contained fully adjudicated service claims from 61 health plans across the US. Inpatient and outpatient diagnoses (ICD-9-CM format) and procedures (CPT-4 and HCPCS formats), as well as standard and mail order prescription records, are included in the data set. Reimbursed payments and charged amounts are available for all services rendered, as well as dates of service for all claims. Additional data elements include demographic variables (e.g., age, gender, geographic region), product type (e.g., HMO, PPO), payor type (e.g., commercial, self-pay), provider specialty, and start and stop dates for plan enrollment. All patients who met the sample selection criteria specified below were included in the analyses.

### Sample selection

Patients with a diagnosis of rheumatoid arthritis (ICD-9-CM 714.XX) who newly started on infliximab or etanercept between July 1999 and June 2001 were initially selected for inclusion in the study sample. A hierarchical procedure was then implemented to stratify patients into treatment cohorts according to their first utilization of a particular medication at a specific point in time. For example, a patient initially receiving infliximab and later receiving etanercept would be classified as an "infliximab" patient for the duration of the study period. An index date for each therapy was established based on the first occurrence of a claim. Patients with no claims activity for the index therapy for six months prior to the index date were deemed "newly started".

Those patients not continuously enrolled during the six-month pretreatment and 12-month follow-up periods were excluded from all analyses. Additionally, patients must have had at least five infusions or prescriptions for their index medication. Patients 65 and older who were not enrolled in a Medicare "risk" plan (i.e., a commercial plan that agrees to undertake full financial risk for a Medicare beneficiary) were excluded from each of the analyses, as such patients may not have had fully visible utilization and cost values due to coordination of medical benefits. All medical and pharmaceutical claims spanning the period January 1, 1999 to June 30, 2002 were then extracted for eligible patients in the data set.

### Measures

The primary measures of interest in this evaluation were the frequency and economic impact of an escalation in biologic dose. Dose escalation was assessed for patients new to infliximab and etanercept during the study period described above. Infliximab and etanercept doses reported at the third infusion/prescription respectively were considered to be the maintenance dose levels. Subsequent utilization was then examined to determine dose escalation. Dose escalation for patients initiating infliximab was based on at the presence of least two occurrences of an increase in the number of vials reported on the infusion claim. The standard period (as indicated on the prescribing information for infliximab) between infusions following the third infusion is eight weeks. Therefore, patients with two infusions within seven weeks on two or more occasions were also considered to have an increase in dose. Dose escalation for patients initiating etanercept therapy was determined according to a change in the average daily dose (expressed in terms of mg per day); average daily dose was calculated based on data from pharmacy claims, using the following formula:

metric strength(25 mg)*quantity dispensed (in vials)/days supplied

Patients having two or more prescriptions with a higher average daily dose than that reported on their maintenance dose were considered to have an increase in dose. Dose escalation results were reported on an overall basis and stratified by age (<18, 18–34, 35–44, 45–54, 55–64, and 65 years and over respectively), geographic region (East, South, Midwest, West), calendar year of biologic therapy initiation (1999, 2000, or 2001), and quartile of pre-index RA-related costs.

In addition to dose escalation, the demographic and clinical characteristics of the sample also were assessed and stratified among patients who did and did not escalate their dose. Characteristics of interest included age, gender, health plan type, geographic region, physician specialty as of the index date, presence of selected pre-index medications, procedures, and comorbid diagnoses, co-diagnosis of Crohn's disease, and pre-index total (i.e., RA-related and unrelated) healthcare costs.

During follow-up, the numbers of prescriptions or infusions of biologic therapy were tracked, as were the costs of all appropriate medical interventions, inpatient, outpatient, and pharmacy services. Costs were tallied for both RA-related and unrelated services and medications. Costs were deemed to be RA-related based on the presence of a relevant diagnosis, medication claim, or procedure (See Appendix 'Additional file 1' for ICD-9-CM, CPT-4, and GPI drug codes).

### Analyses

Based on the sample described above, a series of analyses were conducted as follows:

1. *Dose Escalation *– compared the rate of dose escalation at one year among patients initiating etanercept or infliximab therapies;

2. *Predictive Model for Dose Escalation *– identified RA patients most likely to experience an increase in dose at one year, isolating specific medical, pharmaceutical and demographic characteristics that served as predictors for patients increasing drug utilization; and

3. *Comparison of Annual Costs *– compared costs between etanercept and infliximab stratified by whether or not the patient escalated their dose.

The proportion of patients escalating dose was compared between patients receiving etanercept and infliximab using a chi-square test or Fisher's Exact Test (for cell sizes less than five). In addition, a multiple logistic regression model was applied to identify characteristics that were most predictive of patients experiencing an escalation in dose (including the index biologic therapy). The selection of predictors for inclusion in the models began with a univariate analysis of each variable to determine the frequency of the observations associated with each treatment cohort. An initial model run was performed using the following variables: age group, gender, region, biologic therapy group, plan type, prescribing specialty, RA-related costs during the six month pre-index period, non-RA related costs during the pre-index period, maintenance dose, dummy variables (1 = present, 0 = absent) for the receipt of other RA related medications during the pre-index period, and selected comorbidities. A stepwise method was employed to produce the final model specification using an "entry" level of α = 0.15 and a "stay" level of α = 0.05.

The amount of costs (reimbursed amounts paid by health plans) for all services previously described were calculated on an annual per patient basis for each cohort. Total RA-related, unrelated, and overall costs during the one-year follow-up period were compared controlling for differences in age, gender, pre-index RA related costs and other appropriate variables between the cohorts. A generalized linear model using a gamma distribution was used to control for these differences.

## Results

### Patient demographics and clinical characteristics

Clinical and demographic characteristics of the study sample (N = 1,548) are presented in Table 1. Overall, approximately one-third of patients (31%) were aged 55 or older. However, infliximab use was more concentrated among older patients, as 37% of patients on this therapy were 55 or older; compared to 27% of etanercept patients. Not surprisingly, females had a greater representation than males in the study sample, accounting for nearly three quarters of the study population (74%). Rates were similar for the infliximab and etanercept groups respectively (76.4% and 72.1%). Most patients in the sample were members of an HMO or PPO product; however, the use of infliximab was much lower in the HMO group compared to etanercept (30% vs. 45% respectively) and substantially higher among PPO patients (49% vs. 35% respectively).

The use of other RA-related medications prior to biologic use differed numerically by treatment group. NSAIDs were used more frequently by etanercept users relative to infliximab (42% vs. 26% respectively), as were Cox-II inhibitors (35% vs. 27% respectively) and leflunomide (26% vs. 19% respectively). The rate of pretreatment methotrexate use was similar among the etanercept and infliximab groups (55% and 56%).

Utilization of joint aspiration procedures was numerically higher during the pre-index period for patients in the infliximab group relative to the etanercept sample (35% and 28% respectively). Additionally, pre-index RA related costs also were somewhat higher among infliximab patients ($3,916 vs. $3,585).

### Dose escalation

Patients who initiated infliximab therapy experienced significantly higher rates of dose escalation during the first year of follow-up relative to patients who were initiated on etanercept (58% vs. 18%; p < 0.001) (Table 2). When stratified by pre-index costs, patients initiating infliximab therapy had consistently higher rates of escalation relative to patients on etanercept therapy, although the rate of dose escalation generally increased with pre-index costs; the rate of escalation at one year for patients with the lowest pre-index costs was 50% for the infliximab cohort compared to 17% for patients initiating etanercept (p < 0.001); corresponding rates were 62% and 21% in the highest cost group (p < 0.001). When stratified by year of therapy initiation, age, and geographic region, rates of escalation were significantly higher among patients initiating infliximab therapy relative to the etanercept cohort across all groups. The rate of escalation increased by calendar year for patients receiving infliximab, but declined among etanercept users. Interestingly, while the rate of dose escalation increased with increasing age in the etanercept group, this rate declined in the infliximab group as age increased (beyond age 35); for example, 66.4% of those aged 35–44 in the infliximab group increased their dose, versus 39.3% in patients aged 65 and older. Finally, rates of dose escalation varied considerably by region, with the highest rates observed in the South and Midwest.

### Predictive model for dose escalation

Modeled dose escalation results for patients who initiated infliximab or etanercept therapy are presented in Table 3. The type of biologic therapy was by far the most significant predictor of dose escalation, as patients starting infliximab were over 6 times more likely to increase dose than patients starting on etanercept (Transformed Odds Ratio [OR] = 6.38; p < 0.0001). Patients who were members of an HMO were less likely to have an increase in dose than those who did not. RA patients with a listed comorbid diagnosis of Crohn's disease were less likely to escalate dose (OR = 0.48; p = 0.0477) than those without, while patients utilizing Cox II therapy were significantly more likely to experience an increase in dose over the course of the year (OR = 1.36; p = 0.0175). Patients in the West were much less likely to experience an increase in dose. Patients in the Northeast were 1.92 times more likely to increase dose (p = 0.0215), while patients in the South and Midwest were 1.87 and 1.89 times more likely to dose escalate (p = 0.0102 and 0.0063 respectively). Age and gender were not significant predictors of dose escalation, although the likelihood of dose escalation increased by 4% with every additional $1,000 of RA-related pretreatment costs.

In an effort to better understand the factors associated with infliximab dose escalation, an additional model was conducted among patients initiating infliximab only. Results are presented in Table 4. In this model, patients who belonged to an HMO were significantly less likely to have an increase in dose at one year relative to patients with other coverage (OR = 0.68; p = 0.0372). Patients utilizing methotrexate during pretreatment were more likely to escalate dose than those without (OR = 1.48; p = 0.0216). There was a trend towards significance in terms of an age effect, as infliximab users between the ages of 35–44 were more likely to escalate dose relative to younger patients (OR = 1.94; p = 0.0682).

### Comparison of annual costs

Overall, costs for patients initiating infliximab therapy were numerically higher than for patients in the etanercept group ($19,144 vs. $13,977). Much of the cost difference was due to the difference in drug costs ($13,470 vs. $10,159 respectively). In addition, infliximab patients had higher costs for physician management visits ($691 vs. $381), ancillary services ($1,511 vs. $866), and hospitalizations ($2,277 vs. $1,322). Use of alternative biologic therapy (i.e., use of etanercept in a patient starting on infliximab and vice versa) was minimal, as illustrated by extremely low average annual costs for these alternative strategies.

Average annual RA-related, unrelated, and total costs are also stratified by whether patients underwent dose escalation in Table 5. Patients in the infliximab group who experienced an increase in dose had significantly higher RA-related costs relative to those who remained at maintenance levels ($20,915 vs. $16,713; p < 0.0001). This difference was primarily manifested in lower pharmacy costs for patients not escalating dose; for example, annual infliximab costs were 60% higher for patients escalating dose ($15,998 vs. $10,000; p < 0.001). Ancillary costs were also higher for patients with an increase in dose ($1,601 vs. $1,387). However, RA-related hospitalization costs were lower for patients who had an increase in dose ($1,516 vs. $3,323).

## Discussion

In an effort to better understand the differences in dosing patterns and costs among RA patients on biologic therapy, a retrospective analysis of pharmacy and medical claims for patients new to biologic therapy was undertaken. Dosing frequency and quantity was examined, as were RA-related costs at one year after therapy initiation.

Dosing guidelines suggest that etanercept patients receive two 25 mg vials a week; the use of higher doses has not been studied. The recommended dosing for infliximab is 3 mg/kg of body weight for the first dose, and then at two and six weeks and every eight weeks thereafter. Patients experiencing an inadequate response may increase dose to 10 mg/kg; or they may receive treatment as frequently as every four weeks [15]. The flexibility in these guidelines appears to be necessary, as infliximab patients in our study were much more likely to experience a dose escalation than patients on etanercept. There also appeared to be a strong relationship between the utilization of Cox-II inhibitors as well as pretreatment RA-related costs and dose escalation, indicating that disease severity may play a role in the decision to increase dose. Recent evidence suggests, however, that the relationship between dose escalation and disease activity is nonlinear. In a recent examination of infliximab and etanercept use in Sweden, improvement in disease activity levels following infliximab dose escalation was similar to that observed among infliximab patients not escalating dose as well as etanercept recipients [16].

Lastly and most importantly, differences in RA-related cost among patients new to infliximab and etanercept therapy ($19,144 vs. $13,977) were manifested mainly in the treatment costs ($13,470 vs. $10,159). Management and ancillary services accounted for most of the remaining difference. The difference in treatment costs may be attributed to the higher rate of dose escalation among the infliximab group. These patients had treatment expenses that were ~60% higher than patients who did not dose escalate ($15,998 vs. $10,000), while infliximab patients who did not dose escalate had costs similar to patients in the etanercept group. There was little difference in drug therapy costs among etanercept patients who experienced an increase in dose and those with no change ($10,427 vs. $10,100). These findings highlight the differences in treatment patterns and associated costs among patients new to etanercept and infliximab.

Our study was subject to some important limitations. First, as this was a retrospective analysis of claims data, results were based on amounts billed to health plans. As a result, the unit of measurement for infliximab is billed whole vials. For example, if 1.2 vials were administered to a patient, 2 vials would be billed to the health plan. Therefore, these results may not reflect the true amount of infliximab utilized and may in fact under- or overstate the rate of dose escalation – for example, a patient who increases from 1.2 to 1.7 vials will be shown to have utilized 2 vials in both instances; in contrast, a patient moving from 1.9 to 2.1 vials will appear as having moved from 2 to 3 vials.

In addition, information regarding body mass and/or patient weight was not available. As stated above, infliximab dosing levels may range from 3 mg/kg to 10 mg/kg. Dosing changes resulting from weight changes alone were therefore undetectable. Also, one method of estimation of dose change for infliximab was based on two infusions within seven weeks on two or more occasions. It is possible that some patients may have had these non-standard queuing times simply as a result of scheduling availability, and not as a result of dose escalation. Clear estimates of dose increase due to increased frequency may only be obtained through a more controlled observational study.

Furthermore, no information is available in this administrative database regarding the reason for dose escalation – lack of efficacy, increase in symptoms, other reasons. Our major focus for this study was therefore to simply document that standard dosing assumptions regarding infliximab may lead to erroneous conclusions regarding its cost, given the high level of escalation seen in this and other studies. In addition, the database lacks clinical detail on levels of disease severity as well as other potentially important variables (e.g., working status) for consideration of the full clinical and economic impact of dose escalation.

As with all retrospective study, we cannot rule out the possibility that differences in disease progression and/or severity between patients who do and do not escalate dose may have influenced our findings. Nevertheless, our results remained statistically significant even after controlling for observable differences between groups, indicating that any selection bias would likely only affect the magnitude, not the direction, of our findings.

Finally, while the data used represent final, adjudicated claims in a health plan setting, it is possible that the data elements used are subject to coding or misclassification error. Nevertheless, if such an error rate exists, it is likely not a systematic phenomenon – that is, there is no reason to expect that coding errors would disproportionately affect the infliximab or etanercept samples in our study.

## Conclusions

Despite the limitations noted above, we believe our study has important implications. While, the results of recent observational studies suggest that both infliximab and etanercept are highly effective in clinical practice [17], our findings suggest that patients with rheumatoid arthritis who initiate infliximab therapy are much more likely to experience an increase in dose over the course of one year relative to patients who initiate etanercept. This increase in dose leads to significantly higher pharmacy costs, as well as increases in many other RA-related medical costs. While there may be clinical factors in the decision to pick one route of therapy administration over another, public and private payers alike should carefully consider the economic implications of coverage decisions when targeting appropriate candidates for anti-TNF therapy.

## Competing interests

All authors were employed by PharMetrics, Inc. at the time of this analysis, which was conducted based on an unrestricted research grant from Abbott Laboratories, Inc. No other competing interests are declared by any author, including stocks or other holdings, other financial interests, or non-financial interests.

## Authors' contributions

TG was involved in the conception and design of the study, oversaw and provided quality assurance on all study output, and drafted the methods and results sections of the manuscript. DS was responsible for the design and conduct of all descriptive and statistical analyses. DO was responsible for the conception and design of the study, drafting of the background, discussion, and conclusions sections of the manuscript, and all formal correspondence regarding the study. All authors read and approved the final manuscript.

## Pre-publication history

The pre-publication history for this paper can be accessed here:



## Supplementary Material

Additional File 1 File consists of ICD-9-CM codes, CPT-4 codes, GPI drug codes to describe the various diagnoses, type of drugs and procedures. Click here for file

## Figures and Tables

**Table 1:** Demographic/Clinical Characteristics (6 month pre-period)

Characteristic	Infliximab (N = 598)	Etanercept (N = 950)
Age (%)		
< 18	0.2	4.1
18 – 34	7.0	8.7
35 – 44	19.9	23.3
45 – 54	35.8	37.2
55 – 64	32.4	25.1
65 +	4.7	1.7
Age		
Mean	50.5	46.6
SE	0.4	0.4
Min	16	2
Max	80	88
Gender (% female)	76.4	72.1
Plan Type (%)		
HMO	29.8	44.8
PPO	48.7	34.5
POS	12.5	16.3
Indemnity	7.5	2.6
Other	1.5	1.7
Geographic Region (%)		
East	8.7	15.7
South	45.0	15.9
Midwest	38.8	57.4
West	7.5	11.1
Physician Specialty (%)		
FP/GP	6.4	3.4
Internal Medicine	3.5	3.8
Rheumatology	52.7	64.1
Other	27.6	18.8
Unknown	9.9	9.9
Infusions/Rxs (Infliximab/Etanercept)		
Mean	7.0	10.4
Median	7.5	11.0
SD	2.1	3.8
Pre-index Therapies (%)		
NSAID	26.3	41.6
Cox-II	27.1	34.5
Other Misc. Anti-inflammatory	1.3	3.2
Gold Compound	2.7	3.3
Methotrexate	56.5	55.2
Leflunomide	19.4	25.9
Other DMARD	20.7	26.6
Other RA related therapy	74.9	78.8
Pre-index Medical Diagnoses (%)		
Osteoporosis	9.2	7.5
Depression	3.8	3.2
Selected Pre-index Procedures (%)		
Joint Aspiration/Injection Procedures	34.6	27.7
Synovectomy	0.7	0.7
Arthroplasty	1.5	0.8
Arthrodesis	0.7	0.9
Arthroscopy	0.8	1.4
Liver Function Tests	42.5	54.5
Urinalysis	31.3	33.8
Hematologic/Serologic Tests	81.3	75.8
Bone/Joint Imaging	33.8	31.5
Centesis Procedures	24.1	20.9
Upper Respiratory Infections	5.7	5.8
Chest X-ray	21.4	17.3
Pre-index Total Healthcare Costs ($)		
Mean	3,916.40	3,585.11
SE	225.46	252.39
Min	60.23	0.00
Max	47,306.12	178,388.26

**Table 2:** Percent of Patients with an Increase in Dose After One Year

	Infliximab		Etanercept		
	Patients with 3+ infusions	Pct with an increase in dose	Patients with 3+ infusions	Pct with an increase in dose	p-value

**Overall**	598	57.9%	950	18.1%	<0.001
**by Pre-period RA Costs**					
Quartile 1 – $0 to $502.89	157	49.7%	230	16.5%	<0.001
Quartile 2 – $502.90 to $975	157	62.4%	230	18.7%	<0.001
Quartile 3 – $975.15 to $176	135	57.8%	252	15.9%	<0.001
Quartile 4 – $1766.22 +	149	61.7%	238	21.4%	<0.001
**by Year**					
1999*	0	0.0%	196	22.4%	<0.001
2000	309	53.1%	602	17.9%	<0.001
2001	289	63.0%	152	13.2%	<0.001
**by Region**					
East	52	53.8%	149	20.1%	<0.001
South	269	60.2%	151	17.2%	<0.001
Midwest	232	57.3%	545	19.4%	<0.001
West	45	51.1%	105	9.5%	<0.001
**by Age Group**					
< 18 Years	1	100.0%	39	33.3%	<0.001
18 – 34 Years	42	50.0%	83	14.5%	<0.001
35 – 44 Years	119	66.4%	221	16.7%	<0.001
45 – 54 Years	214	60.3%	353	17.3%	<0.001
55 – 64 Years	194	54.1%	238	18.9%	<0.001
65 + Years	28	39.3%	16	25.0%	<0.001

*Infliximab dosing for RA was not detectable via HCPCS code until June of 2000

**Table 3:** Dose Escalation Regression Estimates – Inflixim ab and Etanercept Dependent Variables (Increase in dose for Inflixim ab or Etanercept 1 = yes, 0 = no)

Parameter	Level	Estimate	Odds-Ratio	Transformed Odds Ratio	Conditional Probability	p-value
Intercept		-0.3129	0.73	n.a.	n.a.	0.1702
HMO	Yes	-0.2246	0.80	1.00	0.37	0.0826
	No	0.0000	1.00	1.25	0.42	
Treatment	Inflixim ab	0.0000	1.00	6.38	0.42	
	Etanercept	-1.8529	0.16	1.00	0.10	<.0001
Region	West	0.0000	1.00	1.00	0.42	
	Northeast	0.6505	1.92	1.92	0.58	0.0215
	Midwest	0.6382	1.89	1.89	0.58	0.0063
	South	0.6252	1.87	1.87	0.58	0.0102
Pre-Period Non RA Related Costs ('000s)	Yes	0.0350	1.04	1.04	0.43	0.0185
	No		1.00	1.00	0.42	
Crohn's Disease	Yes	-0.7328	0.48	1.00	0.26	0.0477
	No	0.0000	1.00	2.08	0.42	
Cox-IIs	Yes	0.3064	1.36	1.36	0.50	0.0175
	No	0.0000	1.00	1.00	0.42	

**Table 4:** Dose Escalation Regression Estimates – Inflixim ab Dependent Variables (Increase in dose for Inflixim ab 1 = yes, 0 = no)

Parameter	Level	Estimate	Odds-Ratio	Conditional Probability	p-value
Intercept		-0.0732	0.93	n.a.	0.8209
Age Group					
	0–34	0.0000	1.00	0.48	
	34–54	0.6639	1.94	0.64	0.0682
	45–54	0.3808	1.46	0.58	0.2594
	55+	0.0648	1.07	0.50	0.8469
HMO	HMO	-0.3810	0.68	0.39	0.0372
Pre-Index Therapy	Methotrexate	0.3900	1.48	0.58	0.0216

**Table 5:** Total Costs of Care for RA Patients at One Year

	**Infliximab N = 598**		**Etanercept N = 950**		**Infliximab: Dose Escalation vs. No Escalation (p-value)**
	
	**Dose Escalation**	**No Escalation**	**Dose Escalation**	**No Escalation**	
	**N = 346**	**N = 252**	**N = 172**	**N = 778**	
	
**Measure**	**Cost per patient (mean, SE), $**				
**RA Related Costs**	$20,914.98 (631.15)	$16,713.20 (1,315.86)	$14,482.45 (519.97)	$13,865.48 (306.88)	<0.0001
Pharmacy:					
Infliximab	$15,997.65 (413.51)	$9,999.87 (293.82)	$313.88 (175.79)	$197.56 (53.23)	<0.0001
Etanercept	$101.26 (45.32)	$178.18 (63.03)	$10,426.90 (254.66)	$10,099.71 (124.94)	
Other	$1,049.00 (69.20)	$1,045.57 (145.45)	$1,089.33 (88.71)	$1,009.24 (48.61)	
Total Pharmacy	$17,147.90 (418.81)	$11,223.62 (350.97)	$11,830.12 (292.80)	$11,306.51 (136.87)	
Outpatient:					
Management	$635.68 (53.29)	$766.75 (112.25)	$377.98 (29.58)	$382.19 (41.21)	
Emergency Room	$13.85 (4.11)	$12.54 (6.32)	$4.72 (1.81)	$7.09 (1.76)	
Ancillary	$1,601.47 (116.33)	$1,387.33 (118.42)	$842.11 (122.13)	$871.60 (90.63)	
Total Outpatient	$2,251.00 (136.95)	$2,166.62 (176.82)	$1,224.81 (135.67)	$1,260.88 (120.64)	
Hospitalization Costs	$1,516.08 (393.06)	$3,322.95 (1,191.16)	$1,427.52 (411.72)	$1,298.08 (249.13)	
**Non-RA Related Costs**	$5,370.17 (536.68)	$6,104.37 (1,210.40)	$3,954.81 (415.94)	$4,177.57 (281.46)	0.0302
**Grand Total**	$26,285.15 (1,022.19)	$22,817.56 (2,431.57)	$18,437.26 (809.69)	$18,043.04 (496.95)	<0.0001
