# Supplementary material for: Patterns of use, dosing, and economic impact of biologic agent use in patients with rheumatoid arthritis: a retrospective cohort study
Source: BMC Musculoskelet Disord. 2004 Oct 14;5:36. doi: 10.1186/1471-2474-5-36 (PMC526206; doi:10.1186/1471-2474-5-36)
Supplement: Additional File 1 — File consists of ICD-9-CM codes, CPT-4 codes, GPI drug codes to describe the various diagnoses, type of drugs and procedures. [file 1471-2474-5-36-S1.doc]

**Appendix ‘Additional file 1’**

Study Medications (NDC and J-Codes)

| Remicade | J1745 |
| --- | --- |
| Enbrel | NDC 58406042534, 58406042541 |

RA Related Medications (GPI & J-Codes)

|  |  |  |
| --- | --- | --- |
| NSAIDs | 661000* | J1885 |
| COX-2 Inhibitors | 661005* |  |
| Misc. Anti-inflammatory Agents | 661010*  661099* |  |
| Gold Compounds | 6620* | J1600, J2910 |
| Immunomodulator DMARDs | 21300050*  662500*  662800*  994020* | J8610, J9250, J9260  J7502,  J7515, J7516 |
| Anti-malarial DMARDs | 13000010*  13000020* |  |
| Cytotoxic DMARDs | 994060*  21101020* | J7500, J7501  J8530, J9070, J9080, J9090, J9091, J9092, J9093, J9094, J9095, J9096, J9097 |
| Chelating Agents | 99200030* |  |
| Inflammatory Bowel Agents | 52500060* |  |
| Non-Narcotic Analgesics, Salicylates | 6410*  649910* |  |
| Narcotic Analgesics | 6510*  6520*  6599* |  |
| Corticosteroids (Oral or Intra-articular) | 2210* | J0702, J0704, |
|  |  | J1095, J1100, |
|  |  | J1700,J1710, J1720, J3303 |
|  |  | J7509,J1020, J1030, J1040,  J2920, J2930, |
|  |  | J7510, J1690, |
|  |  | J7506, J3302, J3301 |

| Pharmacologic Agents Indicated for Other Diagnoses (GPI Codes) |  |
| --- | --- |
| | SSRIs | 58160040xxxxxx | | --- | --- | |  | 5816006xxxxxxx | |  | 5816007xxxxxxx | |  | 5816002xxxxxxx | | Calcium replacement | 30043xxxxxxxxx | |  | 3004201xxxxxxx | |  | 3005xxxxxxxxxx | |  | 30042065xxxxxx | |  |
|  |  |
|  |  |

Procedural Codes for Interventions (J-Codes and CPT Codes)

| Joint Aspiration/Injection Procedures | 2210xxxxxxxxx AND route = INJECTION  J0702, J0704  J1690, J2640, J2650  J1700, J1710, J1720  J1095, J1100  J3301, J3302, J3303  J1020-J1040, J2920, J2930 | 20600-20610, 32000, 32002, 33010, 33011, 76930 |
| --- | --- | --- |
| Synovectomy |  | 23105, 23106, 24102, 26130,  27054, 26140, 26135, 26145, 25105,  25118-25119, 25115-25116 |
| Arthroplasty |  | 27700-27703,24360-24363, 27132,  27125,27134, 27122, 27130, 27137-27138,  23470, 23472, 26535-26536, 27437, 27438,  27440-27443, 27445-27447, 27486-27487,  26530, 26531, 24365, 24366, 21240-21243,  25447, 25441-25446, 25449, 25332 |
| Arthrodesis |  | 23800, 23802, 24800, 24802, 25800-25830,  26820-26863, 27280-27286, 27580, 27870,  27871, 28705-28760 |
| Arthroscopy |  | 29815-29847, 29850-29898 |

Procedural Codes for Lab Tests (CPT Codes)

| Liver Function Tests | 84460, 80058, 84075, 84078, 84080, 84450 |
| --- | --- |
| Urinalysis | 81000-81005 |
| Hematologic/Serologic Tests | 86430, 86431, 80072, 85651, 85652, 86038,  86039, 86215-86235, 85007-85048 |
| Bone/Joint Imaging | 73000-73225, 73500-73725 |
| Centesis Procedures | 20600-20610, 32000, 32002, 33010, 33011, 76930 |
| Upper Respiratory Infections:  -  Sputum analysis  -  Sputum culture  -  Sepsis, blood culture  -  TB culture  -  TB skin test | 89350  87070-87073  87040  87116  86580-86585 |
| Chest X-ray | 71010-71035 |

Diagnostic Codes for Comorbidities (ICD-9 CM)

| Osteoporosis | 733.0x |
| --- | --- |
| Depression | 311, 309.1, 300.4, 301.13, 296.2x, 296.3x, 296.90, 307.42, 298.0, 296.82, 290.13, 290.21, 290.43 |
| Crohn’s disease | 555.xx |
